# Jejunal varices with extra hepatic portal vein obstruction: A case report

**DOI:** 10.1016/j.ijscr.2021.105964

**Published:** 2021-05-12

**Authors:** Bibek Man Shrestha, Suraj Shrestha, Sanjeev Kharel, Ajay K.C, Sujan Shrestha, Sumita Pradhan, Ramesh Singh Bhandari

**Affiliations:** a Maharajgunj Medical Campus, Institute of Medicine, Kathmandu, Nepal; b Department of GI and General Surgery, Tribhuvan University Teaching Hospital, Kathmandu, Nepal

**Keywords:** Gastrointestinal hemorrhage, Jejunal varices, Portal hypertension

## Abstract

**Introduction and importance:**

Extrahepatic portal vein obstruction (EHPVO) with portal hypertension is rare in children. Intestinal varices as new collaterals accompanying portal hypertension are very rare.

**Presentation of case:**

We report an unusual case of a 12-year-old boy with EHPVO with gastrointestinal bleeding from ectopic jejunal varices, without any gastroesophageal varices.

**Discussion:**

Portal hypertension is the most common cause of EHPVO. Among various ectopic varices, intestinal varices are found distal to the duodenum and present with complaints of hematochezia, melena, or intraperitoneal bleeding. The diagnosis of the EHPVO is aided by imaging investigations like Doppler ultrasound, computed tomography, or magnetic resonance imaging. A multidisciplinary team including gastroenterologists, interventional radiologists, surgeons, and intensivists is crucial in the management of ectopic varices.

**Conclusion:**

Jejunal varices must be considered in the differential diagnosis of gastrointestinal (GI) hemorrhage in patients with a negative source of bleed on upper and lower GI endoscopy.

## Highlights

• Intestinal varices are rarely developed as new collaterals accompanying portal hypertension with EHPVO (Extrahepatic portal vein obstruction).
• Jejunal varices must be considered in patients with a negative source of bleed on upper and lower GI endoscopy.

## 1 Introduction

Extrahepatic portal vein obstruction (EHPVO) in a pediatric population is a rare cause of portal hypertension and often upper gastrointestinal bleeding mainly from the esophageal and gastric varices [1]. Rarely, intestinal varices are developed as new collaterals accompanying portal hypertension with extrahepatic portal obstruction. We report a case of a child with EHPVO who presented with gastrointestinal bleeding from the ectopic jejunal varices without gastroesophageal varices. This case has been reported in line with the SCARE checklist [2].

## 2 Case report

A 12-year boy from the Terai region of Nepal presented to the emergency with multiple episodes of painless fresh blood in the stool for 7 days. The first episode occurred 7 days back when he had a sudden severe urge for defecation and the stool contained approximately 100–200 mL of dark red altered blood with clots with very minimal fecal content. He had been having similar episodes 2–3 times a day. The patient had a similar episode of the passage of black stool one year back for which he did not seek medical treatment and the episode was relieved on its own without any further similar episodes in between. Further, there is no bleeding diathesis in the patient or among the family members.

On examination, he was pale, afebrile, tachycardiac, blood pressure of 100/60 mmHg, respiratory rate-18/min with a SpO2 of 98% in room air. Systemic examination along with a detailed abdominal examination was unremarkable with no splenomegaly/hepatomegaly. On Digital rectal examination, the rectum was empty with no mass palpable but the examining finger was bloodstained.

**Fig. 1:**
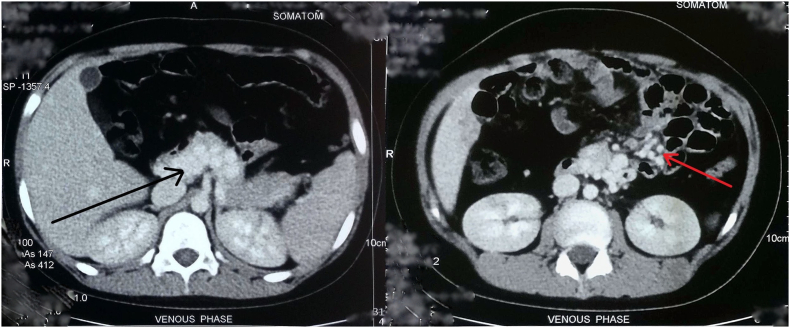
CECT abdomen (axial) showing multiple collaterals around the jejunum (arrows).

**Fig. 2:**
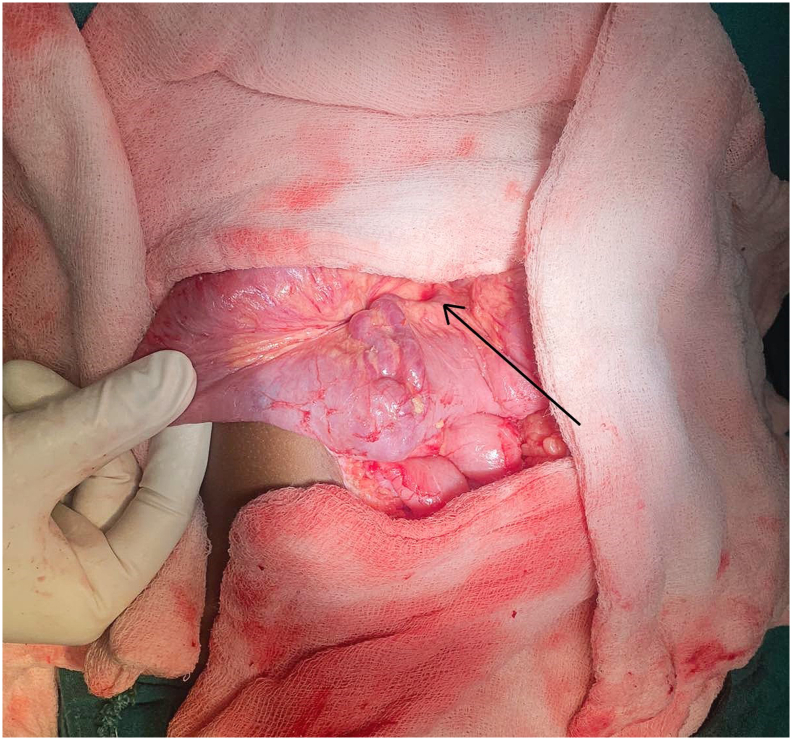
Intraoperative figure showing jejunum with the bleeding point (arrow).

On blood examinations, hemoglobin was 6.5 g/dL, RBC-2.65millions/mm^3^, PCV-21.6, Platelets-359,000/mm^3^, total bilirubin 8 g/L (3–21 g/L), direct bilirubin 3 g/L (0–5 g/L), ALP 171(<306) with other blood parameters within normal range. Owing to low hemoglobin, a blood transfusion was done. For the evaluation of suspected lower gastrointestinal bleed, an upper endoscopy was performed which was normal without any esophageal/gastric varices (Fig. 1). A colonoscopy revealed mucosa covered with blood from transverse to the sigmoid colon. CECT scan of abdomen/pelvis was done that revealed non-visualization of the main portal vein, superior mesenteric vein, and proximal splenic vein with the collateral formation in the intrahepatic region, porta hepatis, peripancreatic region, splenic hilum, and mesentery with collaterals in mesentery in close proximation with the loop of distal jejunum; all features suggestive of EHPVO with jejunal varices (Fig. 2).

**Fig. 3:**
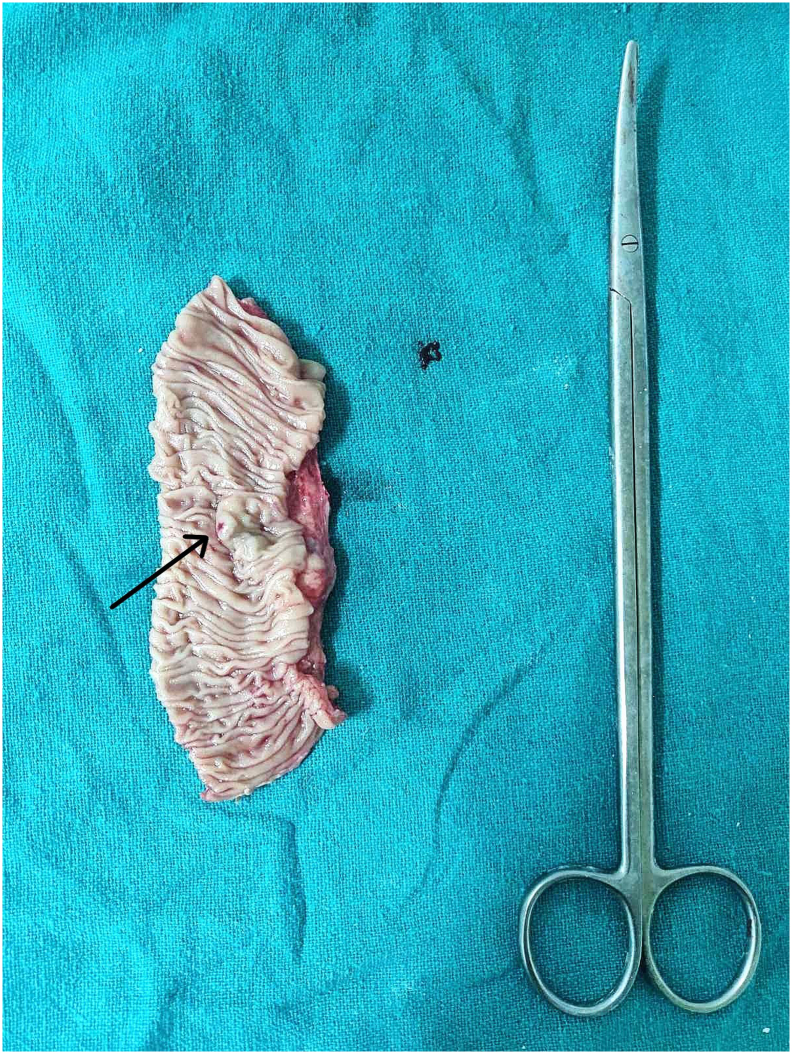
Cut specimen of the jejunum showing bleeding punctum (arrow) from jejunal varix.

The patient was planned for embolization of the bleeding vessels. However, due to the increased risk of delayed perforation and anticipated difficulty to embolize the distal jejunal venous dilations, the patient opted out of the procedure. Thus, the patient was planned for laparotomy with jejunal resection anastomosis. Per-operatively, multiple tortuous collaterals in the mesentery of the small bowel extending up to the wall of jejunum approximately 120 cm proximal to the ileocecal junction along with bleeding site in the jejunum were identified (Fig. 3). Thus, resection and anastomosis of the jejunal segment by the experienced team of gastrointestinal surgeons of Tribhuvan University Teaching Hospital was performed and was well tolerable by the patient. The anastomosis was done in 2 layers with interrupted suture using absorbable suture. As spleen size was normal with no pancytopenia and gastroesophageal varices, splenectomy with shunt procedure was not planned. He passed melena for 3 days in the postoperative period followed by normal colored stool from the fourth postoperative day. The boy was discharged after 10 days of hospital stay when his blood parameters normalized with prophylactic propranolol. At 3 months follow-up, the child was healthy and doing well with no hematemesis, melena, and blood parameters were within normal limits. UGI endoscopy revealed normal findings. At 6 months follow-up, the child was healthy and doing well with no complaints. He is planned for a yearly follow-up.

## 3 Discussion

The cause of EHPVO varies and predisposing factors associated with it are usually detectable along with perinatal events like umbilical catheterization and sepsis, and prothrombotic disorders [3]. In developing countries like Nepal, the most common cause of EHPVO among children is portal hypertension [4]. The neonatal period of our patient was uneventful.

All large pressurized Portosystemic venous collaterals in the abdomen but gastroesophageal region are considered ectopic varices and include collaterals in jejunum, ileum, anal region, uterus, ovaries, and urinary bladder which make up for approximately 5% of all variceal bleeding [5,6]. Abdominal surgery can predispose the patients to the formation of varices in diverse locations like urinary bladder, ovaries, and bare area of the liver occurs in patients with portal hypertension [5,7]. Congenital defects in Portosystemic anastomosis and abnormal vessel structures may also cause ectopic varices [8,9]. Among all ectopic bleeding, Jejunal, and Ileal bleeding are the commonest (approximately 17%) [7]. Along with portal vein thrombosis, cirrhosis and chronic alcoholism may cause jejunal varices [10].

Among various ectopic varices, intestinal varices are found distal to the duodenum with complaints of hematochezia, melena, or intraperitoneal bleeding, and the variceal bleeding is often considered the most serious complication of ruptured ectopic varices [[11], [12], [13]]. Our patient had hematochezia as well as melena with decreased hemoglobin concentration due to chronic blood loss. The diagnosis of the EHPVO is aided by imaging investigations like Doppler ultrasound (US), computed tomography, or magnetic resonance imaging (MRI) which shows obstruction of the portal vein, intraluminal material presents inside, or cavernoma formation [14]. CT scan in our case showed varices in the mesentery extending to the loops of the jejunum. The management of bleeding varices depends on the underlying cause, the expertise of the attending physician, site of hemorrhage, clinical presentation often with collective teamwork of gastroenterologists, interventional radiologists, surgeons, and intensivists [15]. Upper gastrointestinal endoscopy should be done for evaluation of patients with symptoms of slight UGI bleeding and hematochezia. CECT may be preferable to colonoscopy if the findings in the endoscopy are inconclusive of the evidence of the source of hemorrhage [15]. Thus, inconclusive findings of both upper and lower endoscopies in patients with liver disease or portal hypertension increase strong suspicion of ectopic varices [5]. Our patient underwent UGI endoscopy followed by colonoscopy and CECT scan for inconclusive results.

In children, endoscopic and surgical treatment of ectopic varices was preferred in past but now minimally invasive percutaneous and endovascular therapies are the mainstay of treatment [13]. Porto-caval shunt, endoscopic sclerotherapy, embolization, and balloon dilatation, and stent placement in the portal vein are non-surgical treatment options for the management of extrahepatic portal venous obstruction [[16], [17], [18]]. It has been reported that endoscopic treatment including sclerosing agents can be used for the treatment of actively bleeding duodenal or jejunal varices or to prevent re-bleeding from focal varices with hemorrhage [19]. The distal location poses a major drawback for endoscopic management [13].

Surgery is often considered a salvage procedure in selected patients when endoscopic and interventional radiological procedures have failed [15]. The enterectomy of the affected area is helpful when there is hypertension and thrombosis of the portal vein but with limited access for angioplasty, as evidenced by enterectomy for bleeding ileal varices [20]. Because of non-visualization of ectopic varices on upper GI endoscopy and denied embolization, our patient underwent resection of the bleeding jejunal segment and anastomosis.

In medical management, beta-blockers are used for primary and secondary prophylaxis of bleeding from gastroesophageal varices. But, the use of these in the long-term management of patients with ectopic varices is yet to be established [5]. Our patient is receiving prophylactic propranolol.

## 4 Conclusion

Management of ectopic variceal bleeding can be challenging owing to the rarity of the condition and difficulty in diagnosis. Jejunal varices must be considered in the differential diagnosis of gastrointestinal hemorrhage in patients with a negative source of bleed on upper and lower GI endoscopy.

## Ethical approval

Not required.

## Funding

None.

## CRediT authorship contribution statement

Ramesh Singh Bhandari (RSB), Sumita Pradhan (SP) and Sujan Shrestha (SS) = Study concept, Data collection, and surgical therapy for the patient

Bibek Man Shrestha (BMS), Suraj Shrestha (SS) and Ajay K.C (AK) =Writing- original draft preparation

BMS, Suraj Shrestha (SS) and Sanjeev Kharel (SK) = Editing and writing

RSB and SP = senior author and manuscript reviewer

All the authors read and approved the final manuscript.

## Guarantor

Bibek Man Shrestha

## Registration of research studies

Not applicable.

## Consent for publication

Written informed consent was obtained from the patients' father for publication of this case report and accompanying images. A copy of the written consent is available for review by the Editor-in-Chief of this journal on request.

## Provenance and peer review

Not commissioned, externally peer-reviewed.

## Declaration of competing interest

None.
